# Altered Nucleotide-Microtubule Coupling and Increased Mechanical Output by a Kinesin Mutant

**DOI:** 10.1371/journal.pone.0047148

**Published:** 2012-10-16

**Authors:** Hong-Lei Liu, Mark A. Hallen, Sharyn A. Endow

**Affiliations:** 1 Department of Cell Biology, Duke University Medical Center, Durham, North Carolina, United States of America; 2 Department of Biochemistry, Duke University Medical Center, Durham, North Carolina, United States of America; 3 Program in Structural Biology and Biophysics, Duke University Medical Center, Durham, North Carolina, United States of America; University of Technology Sydney, Australia

## Abstract

Kinesin motors hydrolyze ATP to produce force and do work in the cell – how the motors do this is not fully understood, but is thought to depend on the coupling of ATP hydrolysis to microtubule binding by the motor. Transmittal of conformational changes from the microtubule- to the nucleotide-binding site has been proposed to involve the central β-sheet, which could undergo large structural changes important for force production. We show here that mutation of an invariant residue in loop L7 of the central β-sheet of the *Drosophila* kinesin-14 Ncd motor alters both nucleotide and microtubule binding, although the mutated residue is not present in either site. Mutants show weak-ADP/tight-microtubule binding, instead of tight-ADP/weak-microtubule binding like wild type – they hydrolyze ATP faster than wild type, move faster in motility assays, and assemble long spindles with greatly elongated poles, which are also produced by simulations of assembly with tighter microtubule binding and faster sliding. The mutated residue acts like a mechanochemical coupling element – it transmits changes between the microtubule-binding and active sites, and can switch the state of the motor, increasing mechanical output by the motor. One possibility, based on our findings, is that movements by the residue and the loop that contains it could bend or distort the central β-sheet, mediating free energy changes that lead to force production.

## Introduction

Kinesin motors hydrolyze ATP to transport vesicles or organelles, assemble spindles or align chromosomes, or disassemble microtubules, regulating cytoskeletal dynamics. The motors do work in the cell by coupling steps of ATP hydrolysis to microtubule binding and release, undergoing conformational changes that produce force. Although the motor mechanism is not well understood, one proposal is that structural changes induced by motor interactions with microtubules are transmitted through the central β-sheet to the nucleotide-binding pocket; thus, the central β-sheet performs an essential role in mechanochemical coupling and force transduction by the motor [1]. This proposal is based on the striking changes in conformation observed in the central β-sheet of a kinesin-14 motor bound to microtubules in different nucleotide states. Distortion or bending of the β-sheet could store and release free energy during different steps of the nucleotide hydrolysis cycle, playing a central role in force generation by the motor [2]. By contrast, an earlier study reported a difference in the orientation and length of helix α4 at the motor microtubule-binding site in crystal structures of the kinesin-3 KIF1A motor bound to ADP or an ATP analogue [3]. The rotational and translational movement of helix α4 between the ADP and ATP-like states has also been observed in motor-microtubule structures determined by high-resolution (8–10 Å) cryoelectron microscopy [4], [5], and has been proposed to be the force-generating conformational change that controls motor-microtubule interactions and drives motor displacements along microtubules [3]. However, the lack of evidence that this movement affects force production and occurs in all kinesin motors has raised uncertainties regarding this proposal [1].

A structural element of the motor that couples microtubule binding to nucleotide binding would be expected to respond to changes in microtubule binding affinity by changes in nucleotide binding affinity. Mutating essential residues of the element could decouple microtubule and nucleotide binding by the motor. Alternatively, the mutants could alter *both* nucleotide and microtubule binding, e.g., by reducing microtubule binding affinity and increasing nucleotide binding affinity, or vice versa. The predicted inverse nature of these effects arises from the properties of the element in coupling these motor functions and transmitting changes from one site to the other – this causes the wild-type motor to bind tightly to microtubules when it releases ADP, or release from a microtubule when it binds ADP. If the structural element is involved in force transduction by the motor, another potential consequence of mutating an essential residue of the element is that the ATP hydrolysis cycle of the motor might be affected, resulting in increased or decreased mechanical output by the motor. Mutants of this type have not been reported previously for the kinesin motors and would provide much needed information regarding mechanochemical coupling and force transduction by the motors.

We tested the hypothesis that loop L7 of the central β-sheet performs a role in mechanochemical coupling of the kinesin motors by identifying an invariant residue of loop L7 and substituting amino acids with different properties to reduce or enhance its interactions with other residues. Analysis of the mutant motors shows that both nucleotide and microtubule binding are altered – the mutants bind more weakly to ADP and more tightly to microtubules than wild type. Inverse effects on nucleotide and microtubule binding are predicted for a structural element of the motor that plays a central role in coupling the two binding sites and transmitting changes from one site to the other. Moreover, two of the mutants hydrolyze ATP faster than wild type and move faster in microtubule gliding assays – the mutations increase mechanical output by the motor. The increased mechanical output by the motor results in the assembly of long spindles with greatly elongated poles in oocytes, which can be reproduced by increased microtubule sliding velocity and crosslinking in spindle assembly simulations. These findings provide the first functional evidence demonstrating that a residue of loop L7 of the central β-sheet plays an important role in mechanochemical coupling and force transduction by a kinesin motor.

## Results

### Identification of an invariant residue in loop L7 of the kinesin central β-sheet that could couple microtubule to nucleotide binding

**Figure 1:**
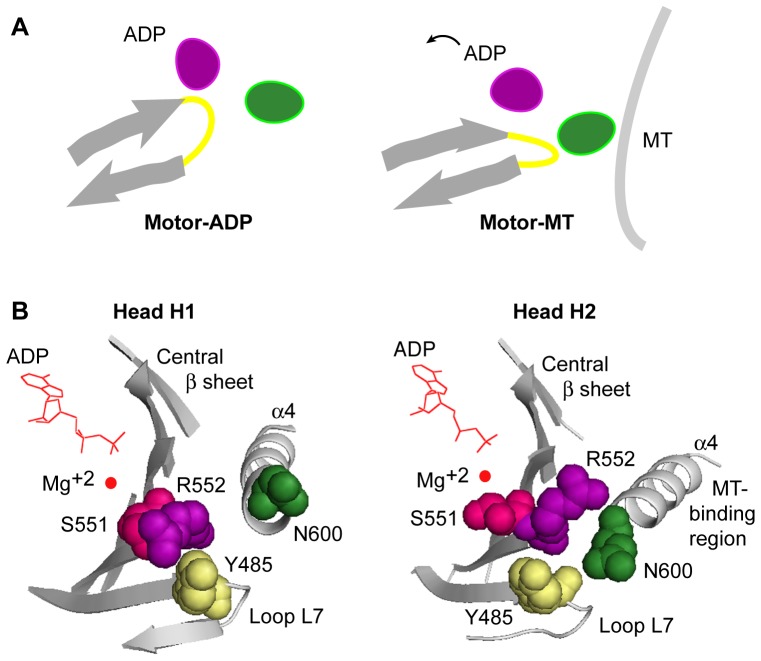
Residue interactions of loop L7 of the kinesin central β-sheet. (A) A loop (yellow) that connects two β-strands (gray arrows) of the central β-sheet could alternately interact with residues that bind nucleotide (ADP, purple oval) or the microtubule (MT, dark green oval) in different nucleotide states, bending or distorting the β-sheet. Mutating a key loop residue could affect both nucleotide and microtubule binding, and alter the stable state of the motor. (B) Two conformations of loop L7 between strands β4 and β5 of the central β-sheet in a stalk-rotated Ncd crystal structure (PDB 3L1C; Movie S1) [12]. In head H1 and both heads of unrotated Ncd dimer structures (PDB 2NCD, 1CZ7), Y485 of loop L7 (space-filled, yellow) touches switch I R552 (space-filled, purple). The adjacent residue, S551 (space-filled, magenta), interacts with the Mg^2+^ coordinating the ADP. In head H2, N600 (space-filled, dark green) at the N terminus of switch II helix ∝4, which interfaces with the microtubule [1], moves towards Y485, and R552 changes in orientation. Images prepared in PyMol [39].

High-resolution (∼10–12 Å) 3D cryo-electron microscopy reconstructions revealed several large conformational changes of a kinesin motor bound to microtubules in different nucleotide states [1]. Among them was a pronounced movement of L7, a hairpin loop that connects strands β4 and β5 of the central β-sheet and undergoes transition into a β-strand in Ncd crystal structures, forming a part of the central β-sheet [6], [7] – L7 moved towards tubulin in the no-nucleotide state, then moved back towards the motor core in the ATP-like state, where it remained in the ADP state. L7 has also been implicated in reconfiguring the active site of the kinesin-3 KIF1A motor, shifting the positions of switch I and II, motifs conserved with G-proteins that show large changes in conformation during nucleotide exchange, causing Mg-ADP to be released [8]. Movements of a loop, such as L7, that connects two strands of a β-sheet could bend or distort the β-sheet in different nucleotide states by alternately interacting with residues that bind nucleotide or the microtubule (Figure 1A). Although it is also possible that L7 moves without causing major conformational changes in the entire β-sheet, correlated nucleotide-specific changes in L7 and the central β-sheet were observed in the cryo-electron microscopy study of the kinesin-14 Kar3 motor described above [1]. This suggests that the backbone motion of Y485 is part of a larger motion of the central β-sheet.

To study this movement further, we identified an invariant residue in loop L7 of the kinesin central β-sheet close to switch I and II. During the ATP hydrolysis cycle, the residue in the kinesin-14 Ncd motor, Y485, alternately interacts with R552 of switch I near the nucleotide-binding cleft and with N600 of switch II helix **α**4 at the microtubule-binding interface (Figure 1B and Movie S1), but is not positioned to directly touch the nucleotide or microtubule. To alter its interactions, we mutated NcdY485 to an acidic, polar or basic amino acid, producing NcdY485E (YE), NcdY485N (YN) and NcdY485K (YK), respectively. The mutants were expressed, purified and tested in biochemical assays for nucleotide and microtubule binding as dimeric Ncd motor proteins containing part of the coiled-coil stalk and conserved motor domain, but deleted for the N-terminal tail, with the corresponding wild-type protein as a control. Mutant and wild-type motor proteins containing almost all of the predicted coiled-coil stalk and motor domain were tested for velocity in microtubule gliding assays.

### NcdY485 mutants release ADP faster than wild type

We first tested the NcdY485 mutants for their ability to bind ATP and release ADP. Single-turnover ADP release assays were performed by incubating the wild-type or NcdY485 mutant motors overnight on ice with fluorescently-labeled mant-ATP to allow the motors to bind to and hydrolyze the nucleotide, then unlabeled ATP was added to induce release of the bound mant-ADP, which was assayed over time.

**Figure 2:**
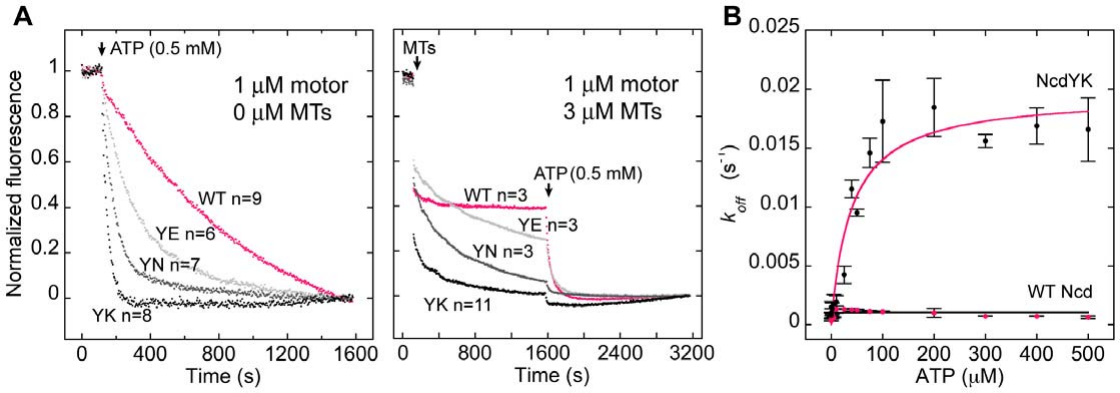
ADP release by the NcdY485 mutants. (A) ADP release without (left) and with microtubules (right). Normalized mean fluorescence versus time after adding ATP (left, arrow) or microtubules followed by ATP (right, arrows) to motor bound to mant-ADP. WT, wild type (magenta). Mutants, YE (gray); YN (dark gray); YK (black). s, seconds. (B) NcdY485K ADP release after adding 0–0.5 mM ATP to 1 µM motor bound to mant-ADP. *k_off_* (s^−1^) per active site vs [ATP]. WT (magenta circles; curve fit, black); NcdYK (black circles; curve fit, magenta). Data points fit to the Michaelis-Menten equation to estimate *V_max_* and *K_M,ATP_*. Error bars, mean±SEM. Assays performed in HEM100.

Fluorescence of mant-nucleotides is increased relative to free nucleotide when bound by the motor; the fluorescence decreases as mant-ADP is released by the motor. Thus, the starting fluorescence levels of the assays provide a measure of ATP binding by the motor. The non-normalized mean data show that the three NcdY485 mutants can bind ATP, although the starting levels are somewhat lower than wild type (Figures S1 and S2). When ATP was added to induce ADP release, all three mutants released ADP much faster than wild type. The averaged data are shown normalized for comparison in Figure 2A (left). ADP release rate constants were estimated by fits of a single exponential decay equation to the data points (Table 1). Comparison of the values revealed that they increase with the mutant in the order YK>YN>YE>WT.

**Table 1:** NcdY485 kinetic parameters.

	WT Ncd	NcdYE	NcdYN	NcdYK
ADP release				
+ATP *k_off_* (s^−1^)	0.00055±0.00008 n = 9	0.00210±0.00009 n = 6	0.0055±0.0002 n = 7	0.0145±0.0009 n = 8
*K_M,ATP_* (µM)	0.7±0.5 n = 2			40±11 n = 3–4
*V_max_* (s^−1^)	0.00103±0.00009 n = 2			0.020±0.001 n = 3–4
MT binding with ADP (fraction bound)	0.20±0.02 n = 4	0.29±0.04 n = 4	0.37±0.04 n = 4	0.6±0.1 n = 4
MT velocity (µm/min)	7.0±0.2 n = 59	7.1±0.1 n = 33	8.8±0.2 n = 32	13.73±0.08 n = 146
ATPase activity				
OD_340_ decrease no MTs (s^−1^)	0.000079±0.000010 n = 4	0.000078±0.000010 n = 4	0.00029±0.00003 n = 2	0.0007±0.0001 n = 2
OD_340_ decrease+1 µM MTs (s^−1^)	0.00020±0.00003 n = 4	0.00017±0.00003 n = 4	0.00034±0.00005 n = 3	0.00107±0.00004 n = 2
*V_max_* (s^−1^)	2.3±0.6 n = 3			4.8±0.2 n = 3
*K_M,MT_* (µM)	17±9 n = 3			1.6±0.3 n = 3

Mant-ADP release rate constants and ATPase rates and rate constants are given per active site. Mean±SEM. MTs, microtubules.

Assays in which microtubules, followed by ATP were added to induce ADP release showed two fluorescence decreases, the first after adding excess microtubules and the second after adding ATP (Figure 2A, right; see Figure S2 for non-normalized data). The decreases for wild-type Ncd were approximately the same magnitude and were interpreted as ADP release by the first and second head of the motor, respectively [9], given that Ncd is a nonprocessive motor that binds by only one head to a microtubule at a time [10]. The mutants also showed two fluorescence decreases, but the first was much larger than the second and consisted of a sharp drop followed by a gradual decrease, rather than just a sharp drop as observed for wild-type Ncd; the remainder of the ADP was released by adding ATP. The initial decrease was interpreted as ADP release by the first head upon binding to a microtubule, together with gradual release of ADP by the unbound head. Rate constants for ADP release were not estimated from the fluorescence decreases because of their rapidity and because the model is not certain [9]; the involvement of a second process during both the first and the second phase means that a single-exponential decay equation is not the correct model to account for ADP release in the assays. Nonetheless, we noted that the amplitude of the initial decrease – the relative amount of ADP released during the first, microtubule-induced phase – increased with the mutant in the order YK>YN>YE>WT (Figure 2A, right), the same order as ADP release without microtubules.

### NcdY485K binds ATP with much lower affinity than wild type

The absolute fluorescence decrease for NcdY485K was only ∼50% as much as wild-type Ncd or the two other mutants (Figures S1 and S2), presumably due to lower nucleotide affinity than the other motors. We tested the possibility that NcdY485K binding affinity for ATP was lower than wild type by performing ATP-induced mant-ADP release assays to measure the Michaelis constant for ATP, *K_M,ATP_*. The NcdY485K *K_M,ATP_* was ∼60-fold higher (mean±SEM, 40±11 µM, n = 3–4) than wild type (0.7±0.5 µM, n = 2) (Figure 2B and Table 1), indicating much lower affinity for ATP; the *V_ma_*_x_ per active site for NcdY485K ADP release (0.020±0.001 s^−1^, n = 3–4) was ∼20-fold greater than wild type (0.00103±0.00009 s^−1^, n = 2), consistent with the much faster ADP release observed in the previous assays. The NcdY485K mutant thus binds ATP with much lower affinity than wild type and releases ADP much faster.

### NcdY485 mutants bind to microtubules more tightly than wild type

**Figure 3:**
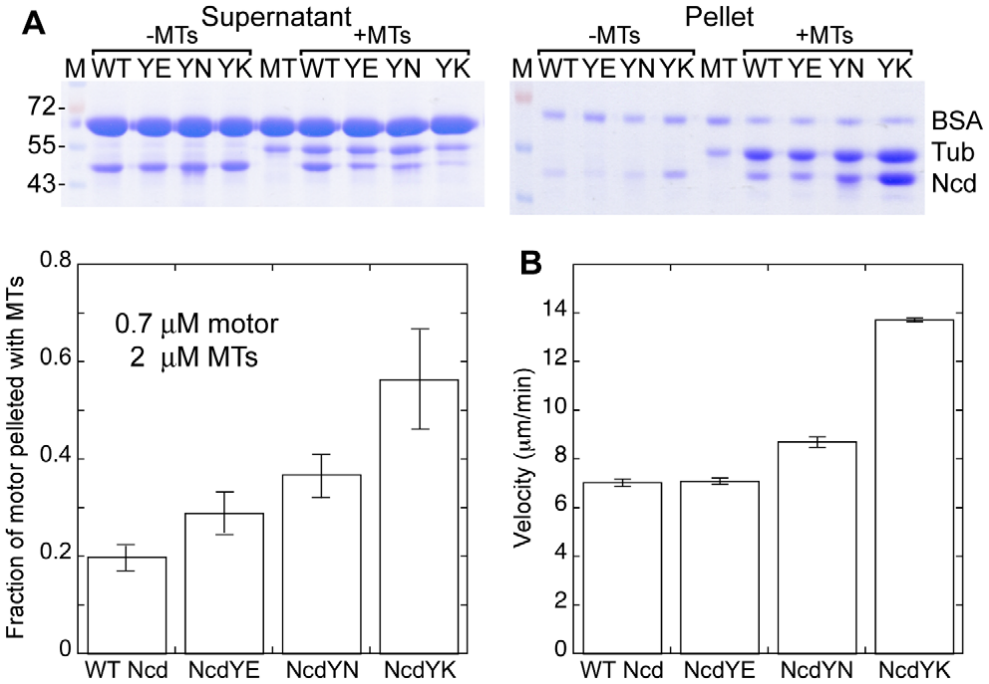
Microtubule binding and motility of NcdY485 mutants. (A) Microtubule binding. Top, supernatants (left) and pellets (right) from assays of motors with added ADP pelleted without or with microtubules. MTs, microtubules; M, M_r_ markers (kDa); Tub, tubulin. Bottom, fraction of motor pelleted with added ADP and microtubules, after correction for motor that pelleted with added ADP but without microtubules. Assays performed in HEM100. (B) Microtubule gliding velocity. Assays in HEM with varying NaCl concentration to optimize motor-microtubule binding and gliding (WT, 50–280 mM NaCl; YE, 100–180 mM NaCl; YN, 250–300 mM NaCl; YK, 250–400 mM NaCl). Error bars, mean±SEM.

We also tested the NcdY485 mutants for microtubule binding in pelleting assays. The motors showed a tendency to aggregate at high motor concentrations and to pellet in the absence of microtubules. Rather than estimating a *K_d_* from microtubules by measuring binding to microtubules at increasing motor concentrations, we performed pelleting assays at a single ratio of excess microtubules to motor (∼3∶1). Relative binding by the motors was determined under the same buffer conditions as the ADP release assays (HEM containing 100 mM NaCl) by analysis of the pelleted motors on the same gel, after correcting for motor that pelleted without microtubules. Strikingly, microtubule binding by the mutants was observed with added ADP (Figure 3A and Table 1) in contrast to the weak binding [11] by wild-type Ncd to microtubules in the presence of ADP. NcdY485K showed the greatest binding – the fraction of the motor that pelleted with microtubules with added ADP (0.6±0.1, n = 4) was 3 times greater than wild type (0.20±0.02, n = 4). The amount of motor that bound specifically to microtubules with added ADP increased with the mutant in the order YK>YN>YE>WT, the same order as ADP release by the motors, indicating a strong correlation between tight microtubule binding and rapid ADP release.

### Microtubule gliding velocity of NcdY485 mutants is faster than wild type

The ability of the NcdY485 mutants to produce force and move on microtubules was tested in ensemble microtubule gliding assays; in addition, these assays provided a further test of microtubule binding by the mutants. Motility was dependent on NaCl concentration in the assays, which, when optimized for each motor, was higher for YN and YK than wild-type Ncd (Table 2), providing further evidence for the high microtubule binding affinity of these mutants. Remarkably, the microtubule gliding velocities of all three mutant motors were as fast or faster than wild type (Figure 3B and Table 1). This is unexpected because altering an invariant residue of the motor is expected to disrupt motor function, rather than not affect or enhance it. The velocities increased with the mutant in the order YK>YN>YE≈WT, paralleling their faster ADP release and higher microtubule binding affinity. NcdY485K showed the fastest microtubule gliding velocity (13.73±0.08 µm/min, n = 146), approximately twice that of wild type (7.0±0.2 µm/min, n = 59). Microtubule leading- and lagging-end velocities did not differ, indicating that the motors were probably not disassembling microtubules at their ends [12].

**Table 2:** NcdY485 mutant motility.

Motor	[NaCl]	Mean velocity*	MTs tracked	Moving MTs/Bound MTs
	mM	µm/min	n	n = total MTs bound
WT Ncd	50 (no precoatƒ)	7.7±0.1	3	1.000 (n = 4)
	50	7.1±0.2	24	0.857 (n = 35)
	250	6.8±0.3	20	0.750 (n = 32)
	280	7.1±0.3	12	0.889 (n = 27)
NcdY485E	100	7.1±0.2	22	0.729 (n = 48)
	180	7.1±0.3	11	0.567 (n = 30)
NcdY485N	250	8.6±0.2	25	0.931 (n = 29)
	300	9.2±0.4	7	0.818 (n = 11)
NcdY485K	250	13.7±0.1	50	0.777 (n = 94)
	300	13.7±0.1	73	0.877 (n = 155)
	400	13.8±0.3	23	0.717 (n = 53)

Ensemble microtubule gliding assays were performed in HEM (10 mM HEPES pH 7.2, 1 mM EGTA, 1 mM MgCl_2_) using the same volume and concentration of anti-GST antibodies to attach the motors to the coverslip. NaCl was added to the assays to the indicated concentrations to optimize motor-microtubule binding and gliding velocity; higher NaCl concentrations than those shown resulted in partially bound or no bound microtubules. Microtubules were not tracked if they were curved or had ends that were unfocused or out of the field, or were recorded <20 s. MTs, microtubules.

* Mean±SEM.

ƒ Unless otherwise indicated, coverslips were precoated with motor before adding a further aliquot of motor for the assay.

### NcdY485 mutants hydrolyze ATP faster than wild type

**Figure 4:**
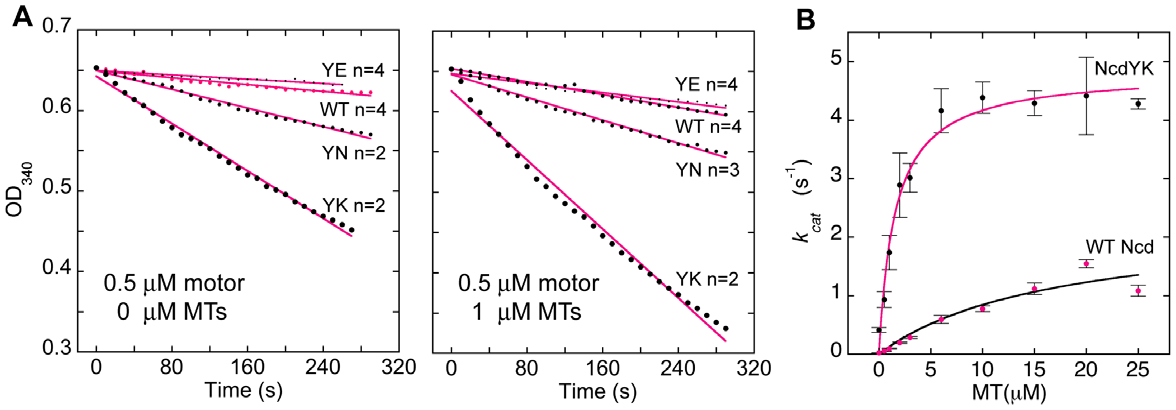
ATP hydrolysis by NcdY485 mutants. (A) Left, basal ATPase activity. Data points adjusted to OD_340_ = 0.6531 (theoretical starting OD of NADH in reaction mix at t = 0) and fit to a line to determine rate of OD_340_ decrease due to ATP hydrolysis. Right, microtubule-stimulated ATPase activity with 0.5 µM motor+1 µM microtubules. (B) NcdYK vs wild-type Ncd ATPase activity. *k_cat_* (s^−1^) per active site vs [MT]; 0.5 µM motor+0–25 µM microtubules (n = 3). Data points fit to the Michaelis-Menten equation to estimate *V_max_* and *K_M,MT_*. Mean±SEM.

Their velocities in motility assays imply that ATP hydrolysis by the NcdY485 mutants is as fast or faster than wild type. We tested this by performing steady-state ATPase assays. Assays of 0.5 µM motor without microtubules and with 1 µM microtubules were performed to determine the relative ATPase activity of the mutants. The results are shown as rates of OD_340_ decrease in the assays per active site of the mutant or wild-type motor (Figure 4A and Table 1). Assays without microtubules showed 5–10 fold faster basal ATPase rates for YN and YK than wild type and a rate for YE that was the same as wild type. Assays with 0.5 µM motor and 1 µM microtubules showed stimulation of the basal ATPase activity of the mutants, giving 2–5 fold faster rates for YN and YK than wild type and a rate for YE that overlapped that of wild type. The rates of ATP hydrolysis increased with the mutant in the order YK>YN>YE≈WT, the same as the gliding velocities.

Assays were also performed with 0–25 µM microtubules to estimate the *V_max_* and *K_M,MT_* for the most severe mutant, NcdY485K, and wild-type Ncd. The assays showed a maximal ATPase rate constant per active site for the NcdYK motor (4.8±0.2 s^−1^, n = 3) that was ∼2-fold higher than wild type (2.3±0.6 s^−1^, n = 3) (Figure 4B and Table 1). The NcdYK *K_M,MT_* (1.6±0.3 µM, n = 3) was ∼11 times lower than wild type (17±9 µM, n = 3), indicating much higher affinity of the NcdYK mutant for microtubules than wild type, consistent with the tighter binding to microtubules by the mutant in pelleting assays.

### Spindles in ncdY485K mutant oocytes are long with greatly elongated poles

**Figure 5:**
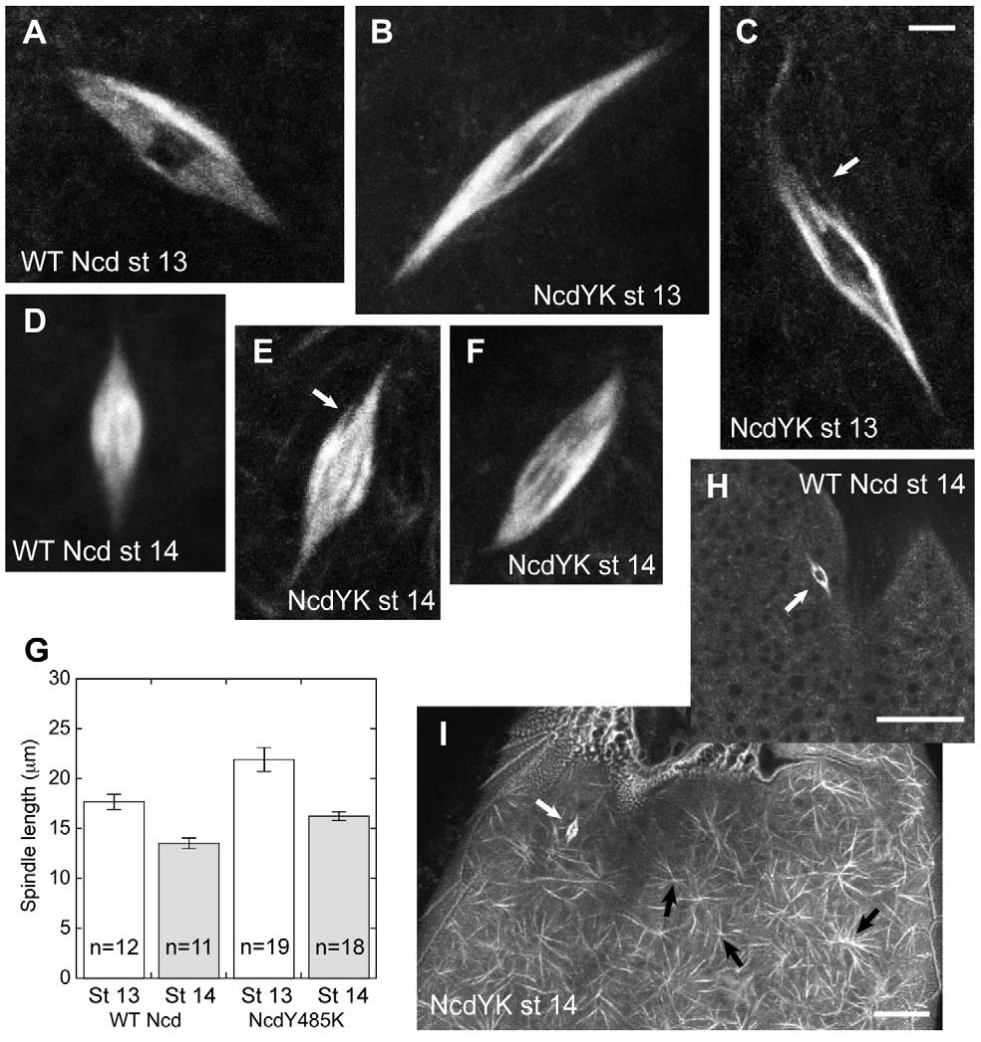
*ncdY485K-gfp* mutant oocyte MI spindles. (A) Wild-type *ncd-gfp* or (B, C) *ncdY485K-gfp* late stage 13 spindles. (D) Wild-type *ncd-gfp* or (E, F) *ncdY485K-gfp* stage 14 spindles. (A–F) Arrows, frayed or spurred spindle fibers. Bar, 3 µm. (G) MI spindle length in wild-type (WT Ncd) or mutant (NcdY485K) oocytes (St 13, late stage 13, white bars; St 14, stage 14, gray bars). Error bars, mean±SEM. (H) Wild-type *ncd-gfp* or (I) mutant *ncdY485K-gfp* stage 14 oocyte cortical region. NcdY485-GFP-decorated cortical microtubules (white) and asters (black arrows) are prominent in *ncdY485K* mutant oocytes. (H, I) White arrows, MI spindle. Bars, 20 µm.

The most severe mutant, NcdY485K, showed ∼11-fold higher microtubule affinity than wild type and ∼2-fold faster velocity in ensemble motility assays. Because of these “improved” functional properties of the NcdY485K mutant over wild type, we also analyzed the effects of the mutant on oocyte spindles *in vivo* (Figure 5A–F). The Ncd motor plays an essential role in meiosis I (MI) spindle assembly in oocytes – mutants cause spindle defects resulting in high frequencies of chromosome nondisjunction and loss, which lead to female sterility and early embryo lethality [13]. Late stage 13 and stage 14 oocytes (n = 44) of *ncdY485K-gfp* females (n = 13) showed mature bipolar MI spindles, many of which were spurred or frayed, or had split poles (n = 19, 43%) (Figure 5C and E), typical of *ncd* mutants [14]. Because the females were *ncd* ^+^, the abnormal spindles indicate dominant-negative mutant effects. Strikingly, the MI spindles of late stage 13 *ncdY485K-gfp* oocytes were frequently longer than normal with long, extended poles (n = 16, total = 24, 67%) (Figure 5B and C); long spindles with greatly elongated poles were also present in mature stage 14 mutant oocytes (n = 3, total = 20, 15%). The frequency of elongated spindles in mutant oocytes was increased by ∼2-fold relative to wild-type oocytes (late stage 13, n = 4, total = 13, 31%; stage 14, n = 1, total = 13, 8%). Overall, MI spindles of late stage 13 (22±1 µm, n = 19) and stage 14 mutant oocytes (16.3±0.5 µm, n = 18) were significantly longer than wild type (stage 13, 17.7±0.8 µm, n = 12; stage 14, 13.5±0.5 µm, n = 11) (Figure 5G).

Wild-type oocytes do not show binding by Ncd-GFP to cortical microtubules (Figure 5H), but many mutant oocytes exhibited NcdY485K-GFP-decorated cortical microtubules (stage 13, n = 5, total = 24; stage 14, n = 10, total = 20) or microtubule asters (stage 13, n = 2; stage 14, n = 1), or both (stage 14, n = 3) (Figure 5I). Ncd bound to cytoplasmic microtubules has not been observed previously – instead, the motor appears to bind specifically to spindle microtubules [15], [16]. The extensive networks of abnormal cortical asters observed in *ncdY485K* mutant oocytes probably arise because the NcdY485K motor binds tightly to microtubules and focuses the microtubule ends, forming asters and arrays that resemble the microtubule arrays formed by the wild-type Ncd motor *in vitro* [17]. The networks of microtubule asters in *ncdY485K* mutant oocytes could explain the origin of the Ncd-associated asters or foci observed in the early stages of MI spindle assembly in *Drosophila* oocytes that migrate towards the condensed meiotic chromosomes and nucleate microtubules to form the spindle [14] – they raise the possibility that the asters arise by Ncd microtubule binding and its minus-end motility.

### Simulations of spindle assembly with tighter microtubule binding and faster microtubule sliding result in longer spindles with elongated poles

Tighter microtubule binding and faster gliding velocity than wild type is expected to increase microtubule crosslinking and sliding rates, respectively. To investigate the role of enhanced crosslinking and sliding in forming the abnormal elongated spindles observed in *ncdY485K* mutant oocytes, simulations of oocyte MI spindle formation were performed using a model we derived previously [18]. In this model, the anastral MI spindle is formed by nucleation of microtubules by Ncd-bound microtubule asters or foci associated with the chromosomes [14], together with microtubule sliding and crosslinking by the Ncd motor. The motor slides microtubules along the long spindle axis to elongate the spindle and form the poles, crosslinks unaligned microtubules with those aligned with the spindle axis, and also crosslinks microtubules associated with different chromosomes.

The two types of crosslinking reflect the fact that crosslinking can either cause elongation of the spindle or stop its elongation, depending on whether the crosslinks align the microtubules roughly parallel to the spindle axis, thus allowing elongation, or roughly perpendicular to it, thus linking the masses of microtubules associated with different chromosomes. Conversely, since the microtubules involved in elongation are crosslinked primarily to other microtubules nearby that are also involved in elongation, these microtubules may not form many crosslinks to microtubules associated with different chromosomes. Thus, the perpendicular microtubules play more of a role in maintaining the normal spindle shape, in which the microtubules associated with all the chromosomes are crosslinked together.

The model uses a set of partial differential equations to predict changes in the length and shape of the spindle during its formation, based on crosslinking and sliding kinetics. The equations represent changes over time in three major populations of microtubules: unaligned microtubules (rho1), microtubules aligned with the long spindle axis (rho2), and those crosslinking different chromosomes (rho3).

**Figure 6:**
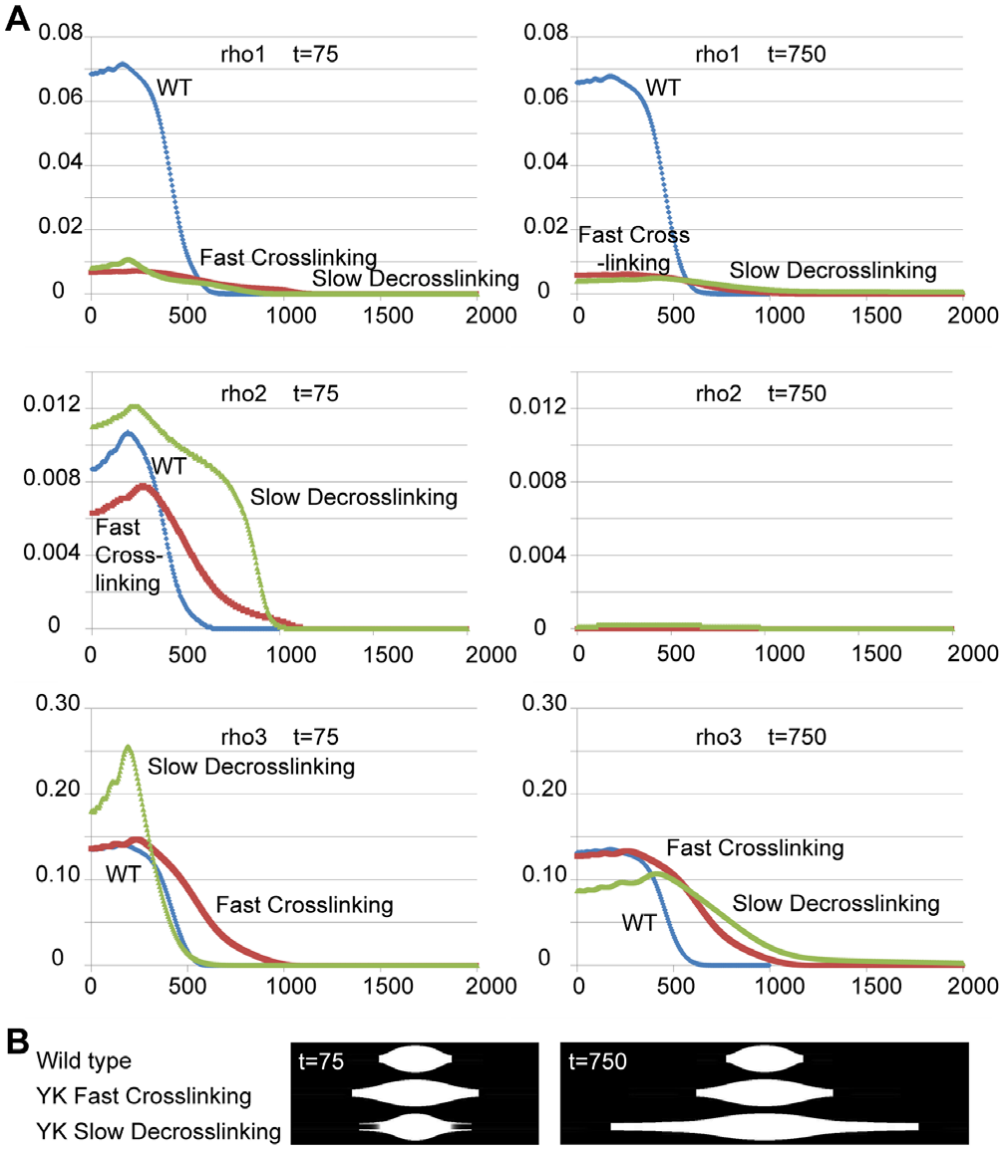
Simulations of NcdY485K mutant effects on MI spindle assembly. (A) Relative microtubule densities (Y axis) versus relative distance from the spindle equator (X axis) in simulations modeling the 11-fold higher microtubule affinity of the NcdY485K mutant as 11-fold faster crosslinking (red) or as 11-fold slower decrosslinking (green), both with 2-fold increased sliding rate to account for faster motility; results for wild-type parameters [18] are shown for comparison (blue). Densities are shown at an early stage of spindle assembly (t = 75, left) and in fully formed spindles (t = 750, right). Top: rho1, unaligned (uncrosslinked) microtubules; middle: rho2, primarily crosslinked microtubules aligned with the long spindle axis, involved in sliding to elongate the spindle; bottom: rho3, microtubules crosslinking different chromosomes, essential for proper spindle shape. (B) Diagrams from the simulations representing spindles at time t = 75 and t = 750.

The population of unaligned microtubules is dominant at the beginning of spindle assembly. The microtubules aligned with the long spindle axis are responsible for sliding, but this population is formed primarily by crosslinking (some are also found at the beginning of spindle assembly). The microtubules crosslinking different chromosomes are needed for the spindle to assume its proper shape. Thus, these different aspects of spindle formation and structure can be quantified using rho1, rho2, and rho3 (Figure 6A).

The overall shape of the spindle is determined by the sum of rho1, rho2, and rho3, which is the total microtubule density. This measure allows the model to predict changes in the length of the spindle, in its formation time, and in the abnormal elongation of the poles (corresponding to microtubule density that drops off more slowly than usual at the poles). Furthermore, an unusually high ratio of rho1 to rho3, particularly at the poles, can be observed in the oocyte spindles as splitting of the poles, or as multiple small spindles if rho1>>rho3 throughout the spindle. We show here by these measures that the model predicts longer spindles and elongated poles when sliding and crosslinking are enhanced, as expected for YK mutant motors. These predictions provide a mechanism for these *in vivo* effects of the mutant.

Kinetic rate constants representing microtubule interactions during alignment, crosslinking and sliding were estimated previously and a set of parameters was identified that represents normal spindle formation by the wild-type Ncd motor [18]. This parameter set (k_a_ = 1, k_d_ = 0.1, k_c_ = 4, k_dc_ = 2, k_r_ = 0.3, and k_e_ = 0.6) was used to represent wild-type Ncd activity in MI spindle assembly [18]. Two new parameter sets were used in simulations to determine the effects of the higher microtubule affinity of the NcdY485K mutant and its faster motility compared to wild type. The increase in mutant microtubule sliding rates was quantified by noting that the *in vivo* microtubule concentration during spindle formation is probably at least 50 µM tubulin [19], which is significantly greater than the *K_M,MT_* of the mutant, and the maximal mutant ATPase rate is ∼2-fold greater than wild type. Thus, the steady-state ATPase rate, which we assume to be proportional to the sliding rate, is ∼2-fold greater for the mutant than wild type as well. In both new parameter sets, the sliding rates (k_r_ and k_e_) were multiplied by a factor of 2 (k_r_ = 0.6, k_e_ = 1.2). Next, to estimate the increase in crosslinking, we assumed that crosslinking mutant motors are bound ∼11-fold tighter than wild-type motors, because *K_M,MT_* is ∼11-fold lower for the mutant. We investigated two possible mechanisms for this (Figure 6). One simulation used an 11-fold increase in crosslinking rates (k_a_ = 11 and k_c_ = 44) and normal rates of breaking of microtubule crosslinks, or decrosslinking rates (k_d_ = 0.1 and k_dc_ = 2), while the other used normal crosslinking rates (k_a_ = 1 and k_c_ = 4) and decrosslinking rates eleven times less than normal (k_d_ = 0.009090909, and k_dc_ = 0.1818181818). Both of these parameter sets are consistent with 11-fold higher microtubule affinity of the mutant, since faster crosslinking and slower decrosslinking are the two mechanisms by which motor affinity for microtubules can affect microtubule crosslinking.

Strikingly, the two parameter sets both generated spindles with features resembling the mutant *ncdY485K* oocyte MI spindles (Figure 6A,B). The spindles in both cases are longer – approximately twice as long or much longer than those observed with the normal parameter set [18] – and the poles are particularly elongated, as observed in many of the mutant spindles. A key difference between the two mutant parameter sets is that spindle formation with faster crosslinking is significantly accelerated relative to the normal parameter set, whereas spindle formation with slower decrosslinking is significantly decelerated. Although crosslinking between microtubules associated with different chromosomes lags slightly behind spindle elongation in all of the simulations, this lag is more pronounced in the slower decrosslinking simulation than the fast crosslinking or normal simulations, leading to markedly split poles during early phases of spindle assembly (Figure 6B, bottom). Spurred or frayed spindles, or spindles with split poles are also observed in many of the mutant *ncdY485K* spindles (Figure 5C and E). These spindles may arise by slow breaking of crosslinks between microtubules parallel to the long spindle axis, which could contribute to spindle elongation, and the lagging behind of crosslinks between the separated poles, causing them to persist. The simulations show that faster microtubule crosslinking or slower decrosslinking due to tighter microtubule binding, together with faster microtubule sliding, result in the formation of longer than normal spindles. In particular, tighter microtubule binding that results in slower decrosslinking is consistent with the observation in *ncdY485K* mutant oocytes of long spindles with greatly elongated poles, many of which are split, spurred or frayed.

The longer than normal spindles shed light on the regulation of spindle length [20]. They imply that MI spindle length is regulated by sliding forces and crosslinking produced by a single motor, as postulated by our model [18], rather than opposing motors – however, the anastral *Drosophila* oocyte MI spindle differs from astral mitotic spindles in its assembly [14], [15] and probably also in its mechanics, given the differences in microtubule organization, polarity and length reported for *Drosophila* and other anastral spindles [19], [21], [22].

## Discussion

We report here the finding that an invariant residue of the kinesin motors present in a hairpin loop, L7, that joins two strands of the central β-sheet, performs a previously unrecognized role in coupling the microtubule-binding surface of the motor to the nucleotide-binding cleft. The residue, Y485 of the kinesin-14 Ncd, is not positioned to interact directly with the nucleotide bound to the cleft or with tubulin, but interacts with other residues that do. Despite the lack of direct interactions with the nucleotide or microtubule, NcdY485 mutants affect both ADP and microtubule binding by the motor, greatly weakening ADP binding, but greatly increasing motor affinity for microtubules.

The Y485 mutants differ from previously reported kinesin mutants that affect the transmittal of changes between the microtubule- and nucleotide-binding site, decoupling the sites [12], [23], [24]. These mutants directly alter residues in the motor nucleotide- or microtubule-binding site, weakening nucleotide binding or enhancing microtubule binding. Instead of these direct interactions, Y485 alternately interacts with residues in both sites to couple them indirectly – it interacts with R552 of switch I in the ADP state, but with N600 of switch II helix **α**4 in the head that releases ADP after the stalk rotates (Figure 1B). The YE, YN and YK mutants were designed to reduce or enhance Y485 interactions with other residues, rather than decouple the two binding sites by fully disrupting the interactions. The mutants alter these interactions and, by doing so, demonstrate the functional importance of the interactions. Changes in Y485 interactions in stalk-rotated Ncd crystal structures are accompanied by release of the bound ADP by the head positioned to bind to a microtubule [7], [12], [25]. The structural changes in Y485 interactions that occur with microtubule binding and ADP release account for the effects of the Y485 mutants on microtubule and nucleotide binding observed in this study.

**Figure 7:**
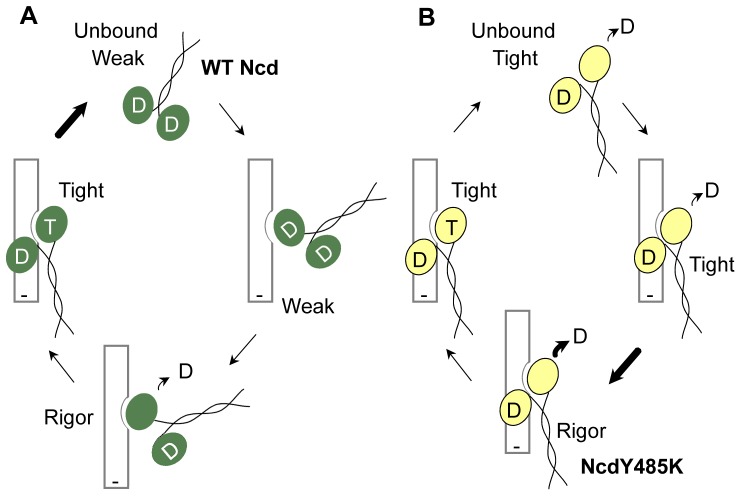
Model of Ncd hydrolysis cycle. (A) Wild-type Ncd exists in a tight-ADP/weak-microtubule binding state in solution (bold arrow) and undergoes cycles of weak-to-tight microtubule binding coupled to nucleotide changes in the head that interacts with the microtubule (D, ADP; T, ATP). (B) NcdY485K and the other two NcdY485 mutants bind tightly to microtubules and may release ADP even when not bound to microtubules; the stable state is the weak-ADP/tight-microtubule binding state (bold arrow), in contrast to wild type. NcdY485K is depicted in a tight microtubule-binding, stalk-rotated conformation like NcdN600K (PDB 1N6M) throughout the cycle [40]; however, the stalk must rotate back to a pre-stroke conformation at least transiently during the cycle for the motor to produce force and support microtubule gliding in motility assays.

The NcdY485 mutants show weak-ADP/tight-microtubule binding, rather than tight-ADP/weak-microtubule binding like wild type. Mutation of Y485 thus alters the stable state of the motor so that it populates a tight microtubule-binding state, rather than a tight ADP-binding state (Figure 7). The inverse effects of the mutants on nucleotide and microtubule binding and the shift in the stable state of the motor implicate loop L7 of the central β-sheet in transmitting changes between the motor microtubule-binding surface and nucleotide-binding cleft. One possibility, based on our findings, is that movements by NcdY485 in different nucleotide states could trigger structural changes in the central β-sheet similar to those observed in the kinesin-14 Kar3 motor, which have been proposed to underlie force transduction by the motor [1].

Weak binding to ADP causes the NcdY485 mutants to release ADP much faster than wild type and alters their nucleotide hydrolysis cycle. Two of the mutants hydrolyze ATP faster than wild type and move faster in ensemble gliding assays. These mutants differ in mechanism from the single-headed kinesin-1 mutants with faster gliding velocities reported recently, which do not show an increase in microtubule-stimulated ATPase activity; instead, the faster velocities are attributed to changes in electrostatic interactions that affect diffusional associations by the motor with microtubules [26]. The NcdY485 mutants show faster gliding velocities than wild type and increased microtubule-activated ATP hydrolysis rates, indicating that their mechanical cycles are faster than wild type. The increased ATPase activity, or greater number of working strokes per unit time, results in greater mechanical output than wild type. The NcdY485K mutant not only moves faster than wild type in gliding assays, but MI spindles in mutant oocytes are longer than normal with greatly elongated poles. Simulations of spindle formation with tighter motor binding to microtubules than wild type and faster microtubule sliding produce long spindles with elongated poles, implying that the longer than normal spindles are caused by the higher microtubule affinity and faster velocity of the mutant observed *in vitro*. Remarkably, one set of simulations results in split poles at early assembly times and mature spindles with greatly elongated poles, resembling the spindles observed in *ncdY485K* mutant oocytes. The visually striking elongated MI spindles highlight the power of dominant gain-of-function mutants [27], [28] in identifying the roles of motors and other proteins in spindle assembly and other cellular functions by their highly noticeable effects.

The faster velocity and greater mechanical output of the NcdY485 mutants implicate the structural element that contains the mutated residue – loop L7 of the central β-sheet – in force transduction by the motor. The invariant tyrosine identified in this study could transmit changes from the motor microtubule-binding interface to the nucleotide-binding cleft, causing structural changes in L7 and the β-sheet. This idea is reminiscent of the proposal by others [29], [30] that the central β-sheet of the evolutionarily related myosin motor transduces force, based on structural changes in crystal structures. Movements by the invariant tyrosine and loop L7 of the kinesin motors could bend or distort the central β-sheet, storing or releasing free energy in different nucleotide states, producing strain that leads to force production [2]. Although it is possible that loop L7 moves, but the entire β-sheet does not change in conformation, movements of the central β-sheet are already apparent in available Ncd crystal structures, even without binding by the motor to microtubules (see Movie S1). Further studies are needed to define the structural changes that occur in loop L7 and the central β-sheet during force production by the motor and the nucleotide hydrolysis steps to which they are coupled.

## Materials and Methods

### NcdY485 mutants

Plasmids to express wild-type Ncd or NcdY485 mutant motor proteins (MGSM-H293-K700) were constructed by conventional methods using *pMW172*, a modified *pET* plasmid [31]. Wild-type and mutant Ncd dimeric proteins contained 54 residues of the stalk and the conserved motor domain; they were deleted for the N-terminal tail and ∼96 residues of the predicted coiled-coil stalk. Proteins were expressed in *Rosetta2 pLysS* bacterial host cells (EMD Millipore Corp.) and purified for biochemical assays by SP-Sepharose ion-exchange chromatography followed by Superose 12 gel-filtration FPLC, as described [32].

### Kinetic assays

Purified proteins were incubated overnight on ice with 4-fold excess mant-ATP (2′(3′)-O-(N-methyl-anthraniloyl)-adenine 5′-triphosphate) prior to performing single-turnover mant-ADP release assays [23]; rate constants were estimated by fits of the data points to a single exponential decay equation. Steady-state ATPase assays using a coupled-enzyme system were performed as described [23], [33]; rates of OD_340_ decrease were determined by fits of a line to the data points after the reaction reached steady state. Assays were performed in HEM100 (10 mM HEPES pH 7.2, 1 mM EGTA, 1 mM MgCl_2_, 100 mM NaCl), except the wild-type Ncd and NcdYK ATPase assays in Figure 4B, which were performed in 100 mM total ionic strength buffer.

### Microtubule pelleting assays

Assays were performed by incubating 0.7 µM purified wild-type or mutant protein with 2 µM microtubules and 0.1 mM Mg·ADP [34], and separating microtubule-bound and -unbound motor protein by centrifugation, followed by SDS-PAGE, as described [23]. Quantitation was performed using ImageJ [35].

### Motility assays

Microtubule gliding assays were performed with motor proteins consisting of glutathione-S-transferase (GST) fused to wild-type or mutant Ncd (K210–K700) containing 137 residues of the ∼150 residue coiled-coil stalk and the conserved motor domain. Lysates of bacterially-expressed GST-Ncd motor proteins were prepared as described [36]. Motor density in the assays was controlled by using anti-GST antibodies to attach the motor to the coverslip surface – the same volume and concentration of antibodies were used in the assays with the wild-type and mutant GST-motors, and the surface was precoated with motor (except for one assay of wild-type Ncd, noted in Table 2) before adding a second aliquot for the assay. This is expected to result in comparable surface densities of the motors in the assays. Initially, both purified motor and lysates were tested under a wide range of motor density and ionic strength conditions. At favorable motor concentrations but lower than optimal ionic strength, bundling of microtubules by the YK mutant was pronounced and microtubule gliding velocity slowed down or stopped, so that microtubules bound to the coverslip surface showed very slow or no motility. Once the concentrations of motor that produced good motility were achieved together with an effective ionic strength that allowed the microtubules to move, the fine-tuning of effects of ionic strength on motor velocity produced relatively small effects, as shown in Table 2. Maximal gliding velocities were obtained by titrating the motors in the assays with varying NaCl concentrations to optimize microtubule binding and gliding velocity (see Table 2).

### Live imaging

MI spindles in oocytes from transgenic *ncdY485K-gfp* flies were imaged in an *ncd^+^* genetic background. Crosses to recover homozygous flies containing the transgene and the *ncd* null mutant, *ca^nd^*, produced no homozygous *ncdY485K-gfp ca^nd^* flies deleted for *ncd*, indicating that the transgene is probably lethal in combination with the null mutant. Live *ncdY485K-gfp ncd^+^* mutant or *ncd-gfp ca^nd^* wild-type [16], [37] oocytes were imaged by confocal microscopy [14] and spindle length was calculated from Z-series stacks [38]. Long spindles with elongated poles were initially identified visually; the frequency of these spindles was determined by measuring spindle length, L, and width, W, and calculating the ratio of length to width. A ratio above the upper limit of a normal distribution of the ratios for wild-type stage 14 oocyte spindles, L/W>5, was used to estimate the frequency of longer than normal spindles in mutant and wild-stage late stage 13 and stage 14 oocytes. The value of L/W>5 is roughly equivalent to the mean of the ratios+2 σ (mean±SD, 3.4±0.9, n = 13). By this criterion, >90% of wild-type stage 14 spindles have poles of normal length.

### Spindle formation simulations

MI spindle formation simulations for the NcdY485K mutant were performed as described previously [18] and in the text. Briefly, the spatial distribution and alignment of microtubules were modelled in MATLAB using partial differential equations with respect to time and distance from the spindle equator (all quantities were unitless), which were discretized with respect to space using second-order finite differences and integrated over time using MATLAB's *ode45* differential equation solver.

## Supporting Information

Figure S1 **Mant-ADP release with added ATP.** Mean fluorescence (a.u., arbitrary units) versus time (s, seconds) after adding 0.5 mM ATP to 1 µM wild-type (WT, magenta, n = 9) or NcdY485 mutant dimeric motor (YE, gray, n = 6; YN, dark gray, n = 7; YK, black, n = 8) bound to mant-ADP at t = 120 s. Normalized curves are shown in Figure 2A (left). The Y485K mutant fluorescence decrease was only ∼50% as much as WT or the two other Y485 mutants.
(TIF)

Figure S2 **Mant-ADP release with added microtubules and ATP.** Mean fluorescence (a.u., arbitrary units) versus time (s, seconds) after adding 3 µM microtubules (MTs) to 1 µM wild-type (WT, magenta, n = 3) or NcdY485 mutant dimeric motor (YE, gray, n = 3; YN, dark gray, n = 3; YK, black, n = 11) bound to mant-ADP at t = 120 s and 0.5 mM ATP at t = 1600 s. Normalized curves are shown in Figure 2A (right). The Y485K mutant fluorescence decrease was only ∼50% as much as WT or the two other Y485 mutants.
(TIF)

Movie S1 **NcdY485 residue interactions.** The movie shows that Y485 of loop L7 (space-filled, yellow), R552 of switch I (space-filled, purple) and N600 (space-filled, dark green) at the end of switch II helix α4 move and undergo changes in interactions between head H2 of a stalk-rotated Ncd structure (PDB 3L1C) and an unrotated Ncd head (PDB 1CZ7); similar changes in interactions are observed between the two heads, H1 and H2, of the stalk-rotated Ncd structure shown in Figure 1B. Animation made in Chimera [41] using default settings of 20 interpolation steps and 60 minimization steps. Small distortional movements of the central β-sheet (ribbon diagram, pale yellow) can be observed. View from the side of head H2 similar to Figure 1B; head H2 is positioned to bind to the microtubule, while the stalk and H1 rotate towards the microtubule minus end.
(MOV)
