# Supplementary figures and images for: Altered Nucleotide-Microtubule Coupling and Increased Mechanical Output by a Kinesin Mutant
Source: PLoS One. 2012 Oct 16;7(10):e47148. doi: 10.1371/journal.pone.0047148 (PMC3473065; doi:10.1371/journal.pone.0047148)

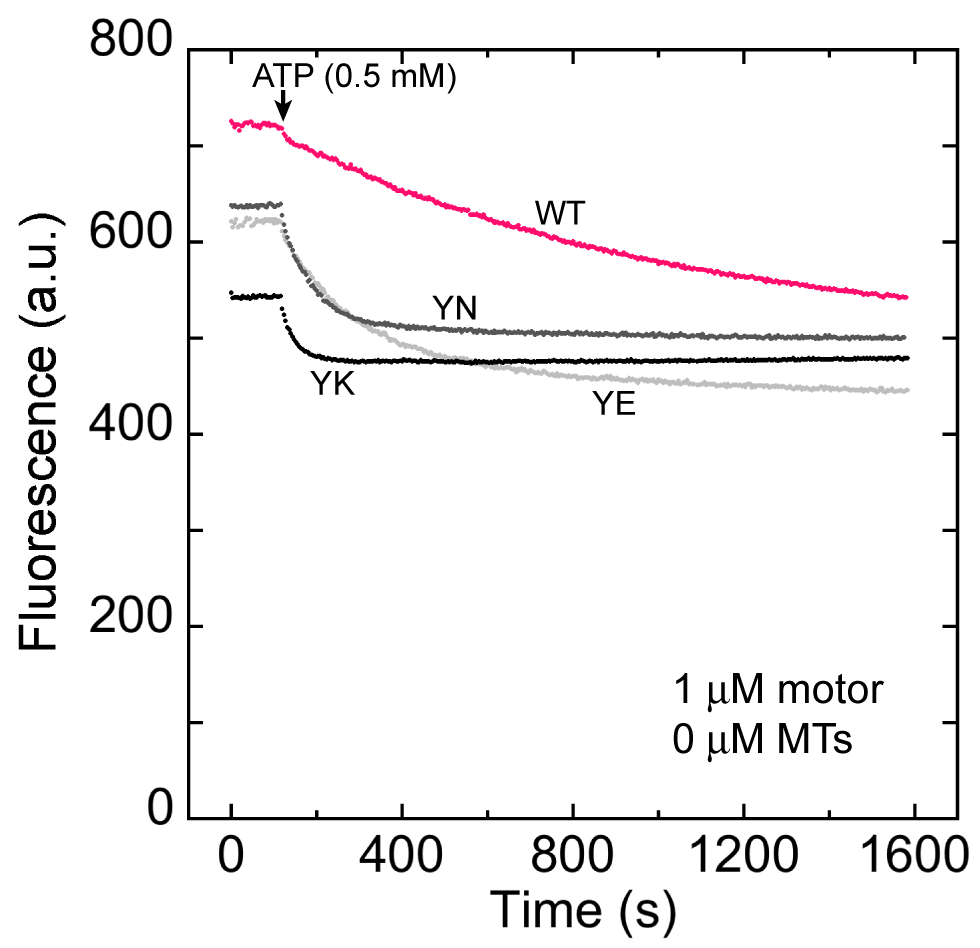

Supplement: Figure S1 — Mant-ADP release with added ATP. Mean fluorescence (a.u., arbitrary units) versus time (s, seconds) after adding 0.5 mM ATP to 1 µM wild-type (WT, magenta, n = 9) or NcdY485 mutant dimeric motor (YE, gray, n = 6; YN, dark gray, n = 7; YK, black, n = 8) bound to mant-ADP at t = 120 s. Normalized curves are shown in Figure 2A (left). The Y485K mutant fluorescence decrease was only ∼50% as much as WT or the two other Y485 mutants. (TIF) [file pone.0047148.s001.tif]

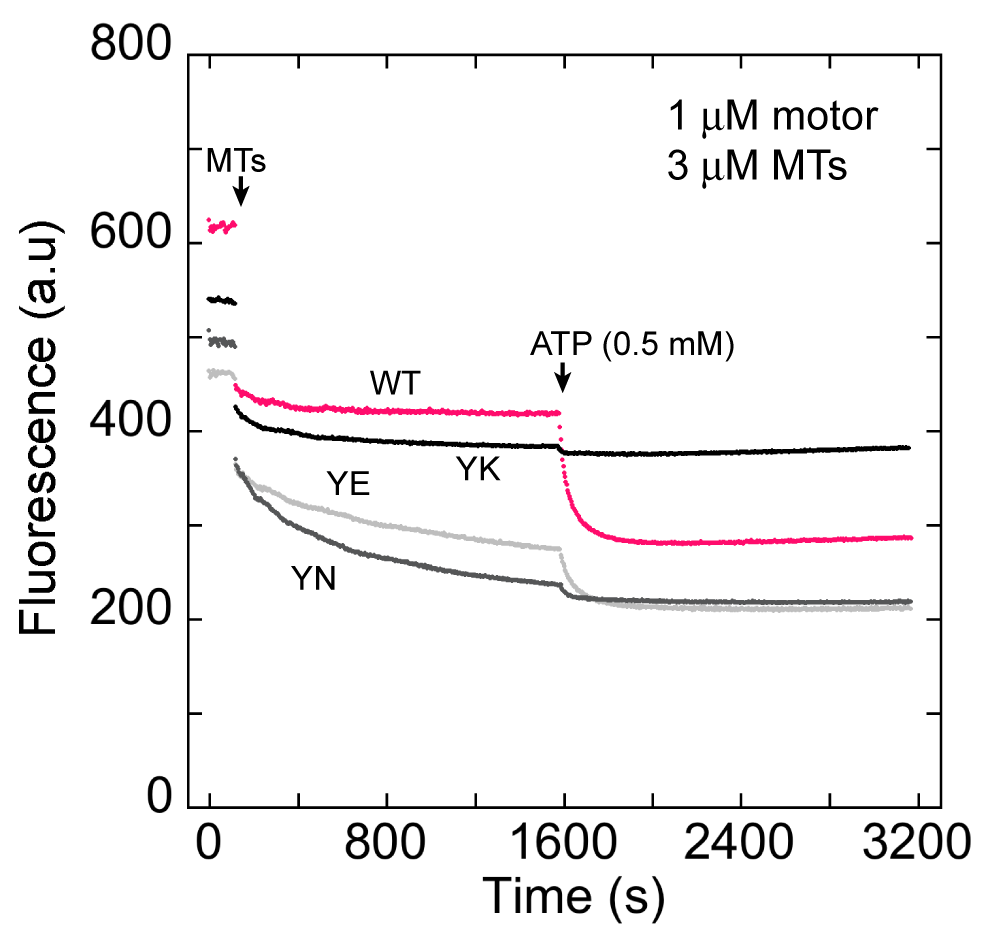

Supplement: Figure S2 — Mant-ADP release with added microtubules and ATP. Mean fluorescence (a.u., arbitrary units) versus time (s, seconds) after adding 3 µM microtubules (MTs) to 1 µM wild-type (WT, magenta, n = 3) or NcdY485 mutant dimeric motor (YE, gray, n = 3; YN, dark gray, n = 3; YK, black, n = 11) bound to mant-ADP at t = 120 s and 0.5 mM ATP at t = 1600 s. Normalized curves are shown in Figure 2A (right). The Y485K mutant fluorescence decrease was only ∼50% as much as WT or the two other Y485 mutants. (TIF) [file pone.0047148.s002.tif]
